# Modeling the effects of war exposure and daily stressors on maternal mental health, parenting, and child psychosocial adjustment: a cross-sectional study with Syrian refugees in Lebanon

**DOI:** 10.1017/gmh.2018.33

**Published:** 2018-12-04

**Authors:** Amanda Sim, Lucy Bowes, Frances Gardner

**Affiliations:** 1 Department of Social Policy & Intervention, University of Oxford, Oxford, UK; 2 Department of Experimental Psychology, University of Oxford, Oxford, UK

**Keywords:** Displacement, mental health, parenting, structural equation modeling, war

## Abstract

**Background.:**

The psychological effects of war trauma are well-documented, but comparatively little research has focused on the mechanisms underlying intergenerational impacts of war and displacement. Specifically, the effects of armed conflict on family processes such as parenting behavior, and subsequent impacts on child psychosocial outcomes, are less understood.

**Methods.:**

This study tests a conceptual model linking past war trauma and current displacement-related stressors to maternal mental health, parenting behavior, and child psychosocial problems. Cross-sectional data were collected in 2016–2017 from a sample of 291 Syrian refugee mothers in Lebanon. We used structural equation modeling to examine associations between war trauma, daily stressors, mothers’ general psychological distress and post-traumatic stress (PTS), negative parenting, and child psychosocial problems.

**Results.:**

Exposure to war-related events was directly associated with maternal PTS and general psychological distress, as well as indirectly via daily stressors. Mothers’ general psychological distress, but not PTS, was directly associated with negative parenting and child psychosocial difficulties. Negative parenting mediated the association between maternal general psychological distress and child psychosocial problems. Model fit statistics indicate that the measurement and structural models provided a good fit to the data.

**Conclusions.:**

Results suggest that the adverse effects of past war trauma and ongoing displacement on refugee mothers’ general mental health can increase the risk of negative parenting behavior, and in turn contribute to poorer psychosocial outcomes for children. Interventions should focus on psychosocial and parenting support for war-affected caregivers, as well as address structural challenges that debilitate caregiver and child mental health.

## Introduction

A growing body of research suggests that contextual factors, particularly caregiver mental health, are crucial in predicting the psychosocial outcomes of war-affected children (Betancourt & Khan, 2008; Tol *et al*., 2013; Meyer *et al*., 2017). For instance, a systematic review examining the effects of armed conflict and terrorism on children aged 0–6 years found that maternal depression and post-traumatic stress (PTS) predicted children's PTS symptoms, behavioral problems, and somatic complaints (Slone & Mann, 2016). Prospective studies with war-affected families in Afghanistan, Pakistan, and Sierra Leone have also demonstrated intergenerational mental health impacts over a 1–4-year period (Panter-Brick *et al*., 2014*b*; Betancourt *et al*., 2015).

While these studies suggest a robust and consistent relationship between caregiver and child mental health in conflict settings, the mechanisms underlying this association are less understood. Hypotheses around causal pathways have focused on the mediating role of parenting quality, largely drawing on evidence of the effects of caregiver mental health on parenting behavior in non-war settings. For example, meta-analytic reviews have found effects of maternal and paternal depression on negative parenting behaviors such as hostility and unresponsiveness (Lovejoy *et al*., 2000; Wilson & Durbin, 2010).

In theoretical and empirical work with war-exposed populations, there has been a strong emphasis on the effects of parental traumatization on parenting behavior. This is likely due in part to the historical predominance of the trauma-focused model of mental health in research on conflict settings, which is centered on exposure to war-related trauma and resulting PTS symptoms (Miller & Rasmussen, 2010). For instance, PTSD as an explanatory model for family violence centers on symptoms such as anger, hyperarousal, and irritability heightening the risk for harsh parenting (Timshel *et al*., 2017). Avoidance-related PTSD symptoms such as emotional numbing and withdrawal may result in lack of emotional attunement and availability. Traumatic experiences may also impair parent–child relationships by disrupting internal attachment representations of the self as caregiver, thereby debilitating parents’ ability to respond sensitively to children's cues (De Haene *et al*., 2010). The association between parental traumatization and harsh parenting has been demonstrated in multiple war-affected contexts including Sri Lanka (Catani *et al*., 2008), northern Uganda (Saile *et al*., 2014), Israel (Halevi *et al*., 2016), and resettled refugees in The Netherlands (van Ee *et al*., 2012).

By comparison, there is less evidence on how other forms of psychological distress (e.g. depression, anxiety) stemming from post- as well as pre-migration stressors may affect parenting behavior. This gap in the literature is striking given evidence of the direct and indirect associations between stressful material and social conditions post-migration, and refugee mental health (Jordans *et al*., 2012; Chen *et al*., 2017). Research with disadvantaged communities in non-war settings suggests that daily stressors such as poverty and discrimination, also ubiquitous in refugee contexts, increase the risk of negative parenting via effects on caregiver mental health (Conger *et al*., 1994; Leventhal & Brooks-Gunn, 2000). In order for interventions to disrupt the intergenerational effects of war-related trauma and adversity, it is vital to analyze the various pathways contributing to harsh parenting and child psychosocial outcomes in conflict settings.

### The present study

This study aims to expand our understanding of how war and displacement-related daily stressors influence child psychosocial wellbeing via effects on maternal mental health and parenting behavior, with the view of informing psychosocial and parenting interventions for Syrian refugee families. The war in Syria has resulted in a displacement crisis of unprecedented scale, with over half of the country's 22 million pre-war population displaced since 2011 (UNOCHA, 2018). In Lebanon alone, there are almost 1 million Syrian refugees registered with the United Nations, approximately one-sixth of the entire Lebanese population (UNHCR, 2018). Using cross-sectional data from 291 Syrian refugee mothers in Lebanon, we test a multiple linkages model in which maternal exposure to war trauma and daily stressors such as poverty and inadequate healthcare are hypothesized to be associated with maternal PTS and general psychological distress, which in turn have cascading and deleterious effects on parenting and child psychosocial outcomes. The hypothesized model builds on previous conceptual and empirical work that includes past exposure to war trauma as well as post-migration daily stressors as predictors of refugee mental health (Miller & Rasmussen, 2010; Jordans *et al*., 2012; Chen *et al*., 2017). Furthermore, we investigate the unique effects of maternal PTS and general psychological distress on harsh parenting and child psychosocial difficulties, in view of the scarce research on the associations between various forms of caregiver distress and parenting and child outcomes among war-affected populations.

Study hypotheses were as follows:

1. Mothers’ past exposure to war trauma would be directly associated with maternal PTS and general psychological distress.
2. The relationship between past war trauma exposure and maternal mental health would be mediated by current experience of daily stressors.
3. Maternal PTS and general psychological distress would be uniquely associated with parental rejection and harsh punishment, as well as child psychosocial difficulties.
4. The relationship between maternal and child mental health would be mediated by parental rejection and harsh punishment.

## Methods

Data for this study were collected in collaboration with the International Rescue Committee (IRC), a non-governmental organization providing economic, educational, and psychosocial services for Syrian refugees and vulnerable host communities in Lebanon. We used baseline data from an evaluation of the IRC's parenting intervention to conduct the analyses reported in this paper. Local IRC staff used community outreach meetings, home visits, and referrals to recruit Syrian caregivers of children aged 2–12 years (the target population for IRC's parenting program) in Akkar, Arsal, Hermel, and Tripoli districts where the IRC had ongoing protection and psychosocial programing. Field staff first described the structure and content of the parenting program and invited caregivers to register their interest. If the individual had a child within the appropriate age range, staff then explained the purpose and procedures of the study by reading aloud from a script. Caregivers were given the option of participating in the parenting program but not the study, and vice versa. Staff obtained verbal consent from caregivers who agreed to participate in the study. Given the vulnerable status of refugees, informed consent procedures emphasized that participation was voluntary and not linked to receiving assistance from the IRC or any other humanitarian organization.

Data collection took place from August 2016 to March 2017. The field team consisted of 16 female and two male staff from the IRC's children's and women's protection programs in the study sites. All interviewers completed training on research ethics, informed consent, and interview skills prior to commencing survey administration using the application Open Data Kit (ODK) on electronic tablets. Pictorial response ratings (e.g. circles of increasing size to depict Likert scales) were used during the interviews to aid participant comprehension. Individual interviews were conducted in Arabic at the participants’ home or the IRC office. In response to adverse events such as participant distress or disclosure of abuse, interviewers followed a safety protocol that included procedures for referral to additional services. In addition, participant endorsement of the item ‘Beat him/her up, that is hit him/her over and over as hard as one could’ was immediately flagged to the IRC child protection staff for follow-up. The study protocol was reviewed by senior IRC staff and approved by the University of Oxford Social Sciences and Humanities Inter-divisional Research Ethics Committee (Ref No. R46541/RE001).

### Measures

Arabic translations of the measures were obtained either from the original developers of the instrument, or from researchers and practitioners who had previously validated or administered the Arabic version in the field. The entire battery of measures underwent an extensive process of field-testing with interviewers and members of the study population, with minor clarifications made to the translations until the field team reached consensus on accuracy and comprehensibility of all the items.

### Sociodemographic information

Sociodemographic data such as maternal age and education, length of displacement, shelter type, and income were collected using items from the United Nations Vulnerability Assessment of Syrian Refugees in Lebanon, an annual household survey conducted with Syrian refugees (UNHCR *et al*., 2015).

### Past war trauma

The Traumatic Events Checklist, adapted from the Harvard Trauma Questionnaire and Gaza Traumatic Event Checklist for use in conflict settings, contains 17 yes/no items assessing experience of war events such as ‘Been forcibly separated from your family’ (Panter-Brick *et al*., 2009). Item scores were summed to obtain an overall measure of mothers’ war trauma exposure.

### Current daily stressors

Mothers’ current experience of daily stressors was assessed with the Humanitarian Emergency Settings Perceived Needs Scale (HESPER) (World Health Organization & King's College London, 2011). HESPER consists of 21 items assessing perceived physical, social, and psychological needs in humanitarian settings (e.g. ‘Do you have a serious problem because you do not have an adequate place to live in?’). The Arabic language version was validated with Iraqi refugees in Jordan and demonstrated excellent, test–retest reliability, and internal consistency (Jordans *et al*., 2012). In this study, one item from the original measure (‘Do you have a serious problem because you have been displaced from your home country, city or village?’) was omitted as all participants were refugees. One item related to legal status was added based on qualitative research suggesting that lack of legal documentation was a source of stress among refugees in Lebanon (Sim *et al*., 2018). Item responses ranged from 0 (*No serious problem*) to 2 (*Very serious problem*) (*α*  = 0.89).

### Maternal PTS

The Post-Traumatic Stress Disorder Checklist-Civilian Version (PCL-C) (Weathers *et al*., 1993) consists of 17 items assessing symptoms of PTS in the last 30 days (e.g. ‘How much were you bothered by repeated, disturbing memories, thoughts, or images of the stressful experience?’). The Arabic translation of this measure was obtained from the International Medical Corps, a humanitarian organization providing mental health services to Syrian refugees in Lebanon. Item responses ranged from 0 (*Not at all*) to 4 (*Extremely*) (*α*  =  0.89).

### Maternal general psychological distress

The 21-item Depression Anxiety and Stress Scale (DASS) Short Form was used to evaluate symptoms of psychological distress in the last 30 days (e.g. ‘I couldn't seem to experience any positive feeling at all’) (Lovibond & Lovibond, 1995). The Arabic version of DASS-21 has been validated with Arabic-speaking immigrants to Australia (Moussa *et al*., 2016.) Item responses ranged from 0 (*Did not apply to me at all*) to 3 (*Applied to me very much, or most of the time*) (*α*  =  0.91).

### Negative parenting

The Parental Acceptance Rejection Questionnaire (PARQ) Short Form consists of 24 items assessing parental warmth, hostility, and indifference or neglect (e.g. ‘I see my child as a big nuisance’) (Rohner & Khaleque, 2008). A meta-analysis of the reliability of the PARQ using data from 51 studies in eight countries found that Cronbach's *alpha* coefficients exceeded 0.70 in all groups, effect sizes were homogeneous across groups, and convergent and discriminant validity were demonstrated (Khaleque & Rohner, 2002). Positive items were reverse-coded to achieve an overall measure of parental rejection. Item responses ranged from 0 (*Almost never true*) to 3 (*Almost always true*) in the last 30 days (*α*  =  0.79).

We used eight items from the Discipline Module of UNICEF's Multiple Indicator Cluster Survey (MICS) to assess mothers’ use of harsh verbal and physical punishment in the last 30 days. The MICS has been implemented in over 100 countries, and was recently administered as part of a representative household survey of Syrian refugees in Lebanon (UNHCR *et al*. 2015). Items include ‘Shouted, yelled, or screamed at him/her’ and ‘Hit or slapped him/her on the face, head or ears’ (UNICEF, 2016). Responses ranged from 0 (*Never*) to 4 (*Almost all the time*) (*α*  = 0.78).

### Child psychosocial difficulties

We used the Strengths and Difficulties Questionnaire (SDQ) to obtain mothers’ report of child psychosocial difficulties (e.g. ‘Often loses temper’) (Goodman, 1997). The Arabic version of the SDQ has been validated for use in epidemiological and clinical studies (Alyahri & Goodman, 2006). Item responses ranged from 0 (*Not true*) to 2 (*Certainly true*) of the child in the last 30 days (*α*  =  0.68).

### Data analysis

We used IBM SPSS Statistics 24 to conduct descriptive and exploratory factor analyses. To examine direct and indirect effects of war trauma exposure and daily stressors on maternal mental health, parenting behavior, and child psychosocial outcomes, we performed structural equation modeling using Mplus 8 (Muthén & Muthén, 1998). We used parceling to create latent variables for all constructs in the model, with the exception of war trauma exposure which was represented by a total sum score. Parceling refers to the technique of creating aggregate-level indicators consisting of the sum or mean of a subset of items (Little *et al*., 2002). Parceling can have psychometric and modeling advantages over the use of individual items or scale scores in structural equation modeling, such as higher reliability, lower likelihood of distributional violations, and fewer parameter estimates (Little *et al*., 2002, 2013; Coffman & MacCallum, 2005). While not without controversy, the view among many methodologists is that parceling, when thoughtfully and carefully applied, can be a useful and appropriate tool when the goal is to understand the relations among different constructs (Little *et al*., 2013).

As the measures used in this study have not been previously validated with the Syrian refugee population, the dimensionality and factor structure of the items when used in this particular context were unknown. Hence, we first performed exploratory factor analysis with oblique rotation to assess the dimensionality of the items as per guidance by Little *et al*. (2002, 2013). We examined eigenvalues, scree plots, and theoretical cohesiveness of the resulting item groupings to assess the factor structure for each measure. Results from the exploratory factor analyses suggested that all item sets were multidimensional, and that in general factor structures did not correspond to established subscales that had been validated with other populations. Given the multidimensional nature of the items, parcels were created using the domain representative approach (Little *et al*., 2002, 2013). With this method, parcels are created by randomly combining items from different dimensions into item sets, thereby ensuring that each parcel is equally representative of all the facets present within the construct (Kishton & Widaman, 1994). The parcels are then used to create the latent variables in the structural equation model.

Models were estimated using full information maximum likelihood (FIML), which uses all available information from the observed data in the analyses. The percentage of participants with missing data was 14%. All parameters were estimated using the bootstrapping procedure with 5000 bootstrapped samples to deal with non-normally distributed variables. We used the following goodness-of-fit indices to evaluate model fit: comparative fit index (CFI, CFI ⩾ 0.95); root mean square error of approximation (RMSEA, RMSEA < 0.06); and, standardized root mean square Residual (SRMR, SRMR < 0.08) (Hu & Bentler, 1999). The MODEL INDIRECT command within Mplus was used to calculate direct and indirect effects. Maternal education, and child sex and age, were included as covariates in the structural equation model based on prior research suggesting that mothers with less education tend to exhibit more negative parenting behaviors, and boys and younger children tend to experience more harsh discipline (UNICEF, 2010; Lansford *et al*., 2015).

## Results

### Sample characteristics

Characteristics of the sampled mothers and the index child on which they reported are summarized in Table 1. At the time of data collection, participants had been displaced in Lebanon an average of 2–3 years. The mean age of mothers was 31.83 years (s.d.  =  8.18) and 53% had completed secondary school. Approximately half of the sample (56%) lived in an informal tented settlement and one-third (31%) in a rented apartment (both individual and shared families), with the remainder in one-room structures. Only 12% of mothers had worked for income in the last 30 days. Participants reported lacking food or money to buy food an average of 13.73 days (s.d.  =  7.87) in the previous month.

**Table 1.** Sample characteristics.

	Mothers (*n* = 291) M (s.d.)	Scale score range
Demographics		
Age (years)	31.83 (8.18)	–
Children under 18	3.36 (1.50)	–
Age of index child	7.44 (3.23)	–
Sex of index child, female *n* (%)	135 (46.4)	–
War trauma (*N* lifetime events)	7.21 (3.48)	0–17
Daily stressors	25.72 (9.02)	0–63
Maternal psychological distress		
Depression Anxiety Stress (DASS)	32.16 (13.18)	0–63
Maternal traumatic stress		
Post-traumatic stress (PCL)	33.41 (12.64)	0–68
Parenting behavior		
Parental rejection (PARQ)	20.78 (9.00)	0–72
Harsh punishment	9.47 (5.80)	0–32
Child psychosocial difficulties		
SDQ total difficulties	16.59 (5.48)	0–40

Data are mean (s.d.), unless otherwise indicated.

### Measurement model testing

**Fig. 1.**
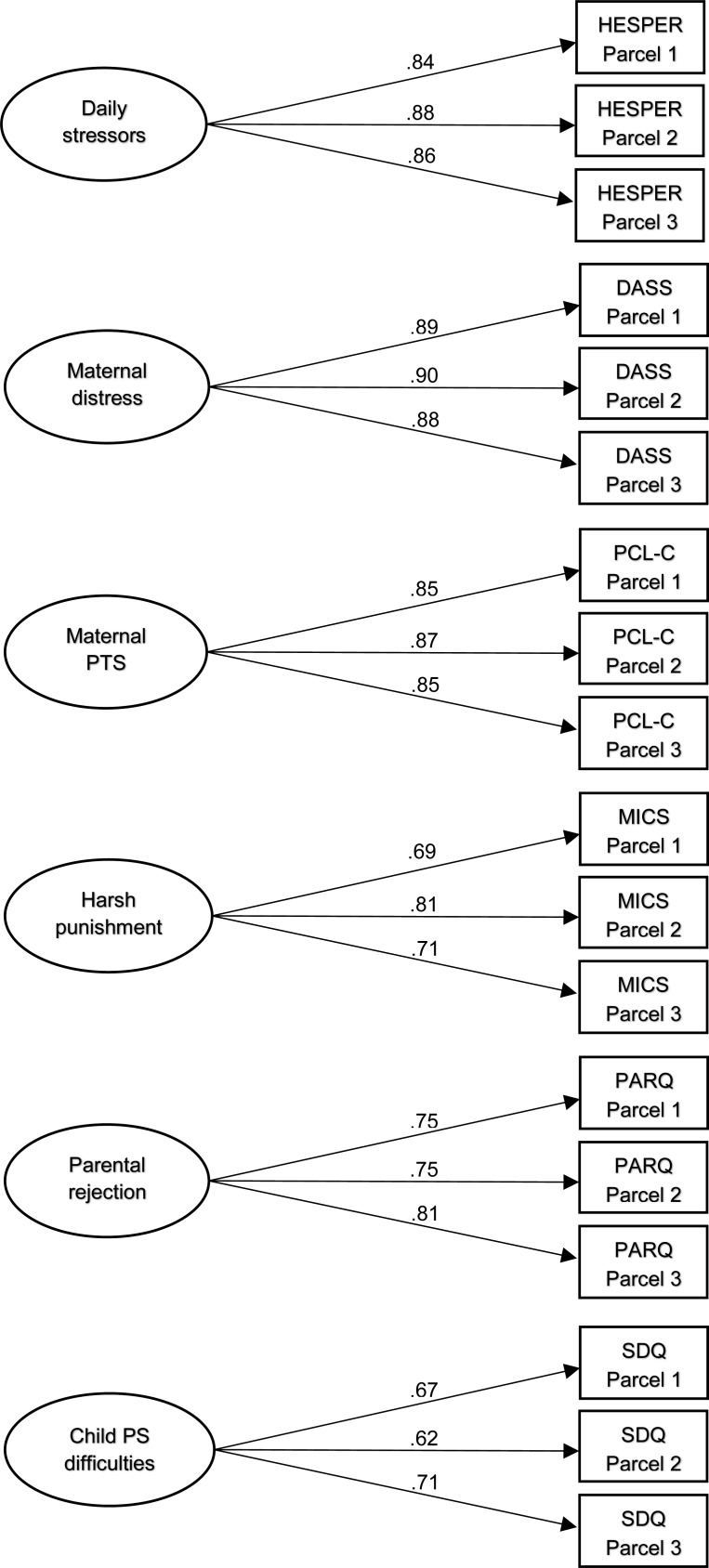
Baseline measurement model with standardized factor loadings. All factor loadings significant at *p* < 0.001. Model fit statistics: χ^2^ [120] = 183.87, *p* < 0.001; CFI = 0.98; RMSEA = 0.04 (90% CI 0.03–0.06); SRMR = 0.04.

We first examined the measurement model with confirmatory factor analysis to establish the latent constructs of daily stressors, maternal psychological distress, maternal PTS, harsh punishment, parental rejection, and child psychosocial difficulties. Model fit indices showed that the baseline measurement model was a good fit with the data: χ^2^ [120]  =  183.87, *p* < 0.001; CFI  =  0.98; RMSEA  =  0.04 [90% confidence interval (CI) 0.03–0.06]; SRMR  =  0.04. All factor loadings were above 0.6 and statistically significant (*p* < 0.001). Correlations between latent variables were all statistically significant (*p* < 0.001) except for daily stressors with parental rejection (see Table 2, Fig. 1).

**Table 2.** Latent variable correlations.

		1	2	3	4	5	6
1	Daily stressors	–	0.45***	0.42***	0.16**	0.08^ns^	0.35***
2	Maternal psychological distress		–	0.70***	0.31***	0.28***	0.56***
3	Maternal PTS			–	0.16**	0.13*	0.35***
4	Harsh punishment			–	–	0.66***	0.50***
5	Parental rejection				–	–	0.48***
6	Child psychosocial difficulties						–

***p < 0.001, **p < 0.05, *p < 0.1, ^ns^*p* ⩾ 0.1.

### Structural model testing

We used structural equation modeling to test the hypothesized pathways between constructs. War trauma exposure had a direct effect on maternal psychological distress (*β*  =  0.36, *p* < 0.001) and maternal PTS (*β*  =  0.45, *p* < 0.001). Daily stressors also had a direct effect on maternal psychological distress (*β*  =  0.30, *p* < 0.001) and maternal PTS (*β*  =  0.25, *p* < 0.001). In addition, daily stressors mediated the association between war trauma and maternal mental health (*β*  =  0.10, *p* < 0.001 for psychological distress; *β*  =  0.08, *p*  =  0.002 for maternal PTS).

Maternal psychological distress had a direct effect on use of harsh punishment (*β*  =  0.34, *p*  =  0.002) and rejecting parenting behavior (*β*  =  0.36, *p* < 0.001). Maternal PTS, however, did not have an effect on either of these forms of negative parenting (*β*  =  −0.03, *p*  =  0.832 for harsh punishment; *β*  =  −0.14, *p*  =  0.205 for rejection). Similarly, maternal psychological distress (*β*  =  0.45, *p* < 0.001), but not PTS (*β*  =  −0.004, *p*  =  0.973), had a direct effect on child psychosocial difficulties. While the direct effects of parental rejection (*β*  =  0.201, *p*  =  0.09) and harsh punishment (*β*  =  0.205, *p*  =  0.13) on child psychosocial outcomes were nonsignificant, there was a total indirect effect of maternal psychological distress on child psychosocial difficulties via parenting behavior (*β*  =  0.14, *p*  =  0.004). There was no indirect effect of maternal PTS on child psychosocial difficulties via parenting behavior.

**Fig. 2.**
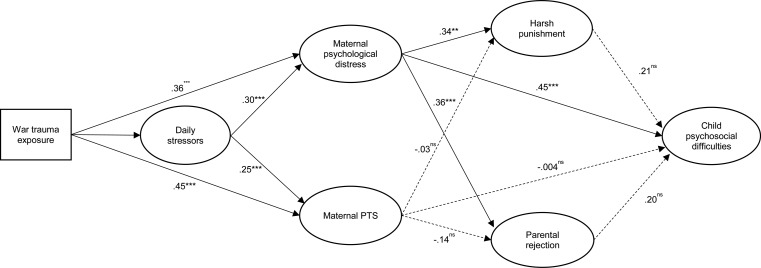
Structural model representing pathways from war trauma and daily stressors to maternal general psychological distress and PTS, and to harsh punishment, parental rejection, and child psychosocial difficulties. Maternal education, and child age and sex, were included as control variables (not shown in figure). All estimates are standardized. Solid lines indicate significant effects and dashed lines indicate nonsignificant effects. ***p < 0.001, **p < 0.05, *p < 0.1, ^ns^*p* ≥ 0.1.

The baseline structural model was a good fit with the data: χ^2^ [186]  =  275.70, *p* < 0.001; CFI  =  0.97; RMSEA  =  0.05 (90% CI 0.04–0.06); SRMR  =  0.04. All analyses controlled for maternal education, and age and sex of the index child (Fig. 2, Table 3).

**Table 3.** Direct, total indirect, and specific indirect effects (standardized regression coefficients and 95% CIs).

	*n* = 291 Std. *β* (95% CI)
Direct effect of war trauma exposure on maternal psychological distress	0.355*** (0.282–0.570)
Indirect effect of war trauma exposure on maternal psychological distress via daily stressors	0.097*** (0.045–0.149)
Direct effect of war trauma exposure on maternal PTS	0.448*** (0.386–0.694)
Indirect effect of war trauma exposure on maternal PTS via daily stressors	0.081** (0.029–0.134)
Direct effect of maternal psychological distress on child psychosocial difficulties	0.445*** (0.237–0.654)
Sum of indirect effect of maternal psychological distress on child psychosocial difficulties via harsh punishment and parental rejection	0.140** (0.044–0.236)
Specific indirect effect of maternal psychological distress on child psychosocial difficulties via harsh punishment	0.069^ns^ (−0.035–0.173)
Specific indirect effect of maternal psychological distress on child psychosocial difficulties via parental rejection	0.071^ns^ (−0.026–0.168)
Direct effect of maternal PTS on child psychosocial difficulties	−0.004^ns^ (−0.207–0.200)
Sum of indirect effect of maternal PTS on child psychosocial difficulties via harsh punishment and parental rejection	−0.033^ns^ (−0.121–0.055)
Specific indirect effect of maternal PTS on child psychosocial difficulties via harsh punishment	−0.005^ns^ (−0.080–0.052)
Specific indirect effect of maternal PTS on child psychosocial difficulties via parental rejection	−0.028^ns^ (−0.089–0.034)

Analyses adjusted for maternal education, and child sex and age.

***p < 0.001, **p < 0.05, *p < 0.1, ^ns^*p* ⩾ 0.1.

## Discussion

This study aimed to represent the processes contributing to the psychosocial outcomes of Syrian refugee mothers and their children. We tested a multiple linkages model in which past war trauma and current daily stressors were hypothesized to be associated with poor maternal mental health, which in turn would be linked to negative parenting behavior and child psychosocial difficulties. Consistent with prior research on the mental health consequences of direct trauma exposure, our results showed that past exposure to war-related events was associated with both maternal PTS and general psychological distress. Furthermore, current daily stressors contributed directly to maternal PTS and general psychological distress, as well as mediated the relationship between war trauma exposure and maternal mental health. Our findings are consistent with the conceptual model elaborated by Miller & Rasmussen (2010), which posits that stressful material and social conditions caused or exacerbated by armed conflict and displacement are a primary pathway through which war exposure impacts mental health. Qualitative research with Syrian refugees in Lebanon has also found that displacement-related daily stressors such as poverty and lack of legal documentation were perceived by refugees as the primary drivers of parental distress and impaired parenting (Sim *et al*., 2018).

Study results also showed a direct link between mothers’ general psychological distress and child psychosocial difficulties, thus contributing to a growing body of evidence on the impact of caregiver mental health on child and youth mental health in conflict settings. A recent study with Syrian parent–child dyads in Turkey, for instance, found that parental psychopathology was associated with child psychosocial problems, even after accounting for children's war trauma exposure (Eruyar *et al*., 2018). Furthermore, our results support extant theory and research positing impaired parenting as one of the primary mechanisms underlying such intergenerational mental health impacts (Fazel & Betancourt, 2017). In our study, mothers’ general psychological distress had direct effects on parental rejection and harsh punishment, and the combined effect of both forms of negative parenting mediated the association between maternal and child mental health. These findings are consistent with a robust body of research from diverse socioeconomic and cultural groups on the negative impact of poor parental mental health on parenting behavior, and subsequent effects of harsh parenting on child psychosocial outcomes (Lee *et al*., 2012; Lansford *et al*., 2014).

Surprisingly, our study did not find a direct association between maternal PTS and child psychosocial problems, or maternal PTS and negative parenting. This contradicts the bulk of evidence on the negative effects of PTSD on parenting and child outcomes, most notably a longitudinal cohort study of refugees resettled in Australia, which found direct and indirect associations between caregiver PTSD and child psychosocial problems via increased harsh parenting (Bryant *et al*., 2018). However, it should be noted that the authors of this study cite as a limitation the lack of assessment of other parental mental health problems such as depression, which might differentially affect parenting and child outcomes. Given the modest sample size and the high correlation between maternal general psychological distress and PTS in the current study (*r*  =  0.70), results showing a null association between maternal PTS, negative parenting, and child psychosocial difficulties should be interpreted with caution. A synthesis of the psychometric properties of the PTSD Checklist demonstrated high correlation with measures of depression and general anxiety, suggesting that it may pick up general negative affect rather than symptoms specific to PTSD (Wilkins, Lang & Norman, 2011). The null effect of maternal PTS in our study may therefore be due to the lack of statistical power given the modest sample size, overlap between measures, and high comorbidity between PTS and depression, anxiety, and stress (Conybeare *et al*., 2012).

### Study limitations

Study results and conclusions should be viewed in light of several limitations. Our sample, while recruited from four districts in Lebanon with a large refugee presence, was not representative and thus results cannot be generalized to the Syrian refugee population as a whole. The relatively modest sample size also limits statistical power to detect potentially small effects. While findings suggest an important relationship between maternal mental health, parenting, and child psychosocial problems, the use of cross-sectional data precludes causal inferences. It is possible, for example, that poor parental mental health may actually cause or exacerbate daily stressors, thus operating in the opposite direction than hypothesized. Determinants of parenting are complex and multidimensional, with intersecting drivers such as parents’ own childhood experience of maltreatment and sociocultural norms (Lansford *et al*., 2015; Fulu *et al*., 2017). While this study goes beyond simple bivariate associations to represent a more nuanced explanation of the processes contributing to child psychosocial outcomes, the model does not account for potential confounding factors including child-driven effects such as child temperament and children's own exposure to trauma and daily stressors. The measures used in this study have not been validated with the Syrian refugee population. Due to the small sample size, we were not able to split the sample into two subsamples, one for exploratory factor analysis and one for confirmatory factor analysis, which would have provided a more stringent test of factorial structure (Wang *et al*., 2013). Finally, data were maternal self-reports only, which increases the risk of bias associated with shared method variance, particularly as parental distress has been found to affect ratings of offspring symptomatology (Smith *et al*., 2001).

The use of prospective, longitudinal designs with a multi-informant assessment strategy will be important for inferring causality and capturing the mental health trajectories of war-affected children and caregivers in future research. Further work on measurement development and validation for the Syrian refugee population is also necessary to be able to discriminate between different dimensions of mental health, specifically to disentangle the potential effects of trauma-specific symptoms such as re-experiencing from those of general distress. Specific outreach to male caregivers is also important to determine if the pathways in the model investigated in this paper operate differently for male and female caregivers, as suggested by evidence from other studies of war-affected families (Palosaari *et al*., 2013; Panter-Brick *et al*., 2014*a*).

## Conclusion

While the direct effects of war on adult and child mental health have been well-documented, research on the pathways through which child psychosocial outcomes are shaped by impaired parental mental health and parenting behavior is lacking. Our study contributes to the growing literature on parenting in war by testing a comprehensive model linking war trauma and daily stressors to maternal mental health, parenting, and child psychosocial adjustment, and by modeling the effects of maternal PTS and general psychological distress on parenting and child outcomes.

Our results have several implications for policy and practice. Given the strong influence of caregiver-related variables, interventions to improve the mental health and psychosocial resilience of war-affected children also need to address caregiver mental health and its impacts on parenting behavior. An ongoing debate among mental health scholars and practitioners concerns the prioritization and sequencing of specialized clinical treatment for PTSD versus interventions for other manifestations of distress, including depression and impaired social functioning (Miller & Rasmussen, 2010; Neuner, 2010). Results highlighting the importance of general psychological distress stemming from multiple pre- and post-migration factors, while preliminary and should be interpreted with caution, suggest that addressing general mental health problems among war-affected caregivers may have wide-ranging, beneficial impacts on overall functioning, and parenting in particular. For example, adopting a common elements treatment approach, in which cognitive behavioral and parenting components can be flexibly combined and delivered by trained paraprofessionals to treat a range of emotional and behavioral problems, may be a feasible and effective intervention strategy for improving caregiver mental health and parenting in conflict settings (Murray *et al*., 2018).

Refugee policies and programs must also address the structural challenges that debilitate caregiver and child mental health, such as inadequate support for basic survival needs, lack of access to quality health and education services, and restrictions on movement and employment (Fazel & Betancourt, 2017; Sim *et al*., 2018). Adopting a multi-level, cross-sectoral intervention approach to reduce or mitigate the impacts of structural and family-related stressors will be instrumental to protecting the psychosocial wellbeing of subsequent generations of Syrian children.
